# Efficacy and safety of Sofosbuvir-containing regimens in patients co-infected with chronic hepatitis C virus and human immunodeficiency virus: a meta-analysis

**DOI:** 10.1186/s12985-018-0934-6

**Published:** 2018-01-19

**Authors:** Guotao Li, Ke Zang, Guoqiang Zhang, Danyan Zhu, Xiaozhao Deng

**Affiliations:** 1 Department of Infectious Diseases, LuoYang Central Hospital Affiliated to ZhengZhou University, Luoyang, Henan 471000 China; 2 HuaDong Research Institute for Medicine and Biotecnics, No.293 Zhongshan East Road, Nanjing, 210002 China

**Keywords:** Sofosbuvir, HCV/HIV co-infection, SVR12, Meta-analysis

## Abstract

**Background:**

The treatment of hepatitis C virus (HCV) in HCV/human immunodeficiency virus (HIV) co-infected patients remains complex. This present meta-analysis evaluated the efficacy and safety of Sofosbuvir (SOF) for treatment in HCV/HIV co-infected patients using the most recent and available data.

**Methods:**

A systematic search of the published data was conducted in PubMed Medline, EMBASE and Cochrane databases. Eligible studies were clinical trials, case-control studies or prospective cohort studies aiming at assessing the efficacy and safety of the SOF-containing regimens in patients co-infected with HCV and HIV. Heterogeneity of results was assessed and a pooled analysis was performed using random effects model with maximum likelihood estimate and 95% confidence intervals (95%CI). Subgroup analysis and assessment of publication bias through Egger’s test were also performed. STATA 13.0 software was used to analyze the data.

**Results:**

Seven studies (*n* = 1167 co-infected patients) were included in this analysis. The pooled estimate of sustained virological response at 12 weeks (SVR12) was 94.0% (95%CI: 92.0%–95.0%). Subgroup analysis showed that the treatment-naïve patients had higher SVR12 compared with patients that were treated before (χ^2^ = 21.39, *P* < 0.01). The pooled incidence of any adverse events (AEs) was 79.6% (95%CI: 77.1%–82.1%). Publication bias did not exist.

**Conclusion:**

The results of this study showed that the treatment response of SOF-containing regimens in patients co-infected with HIV and HCV was satisfied. Attention should be paid to the high rates of AEs.

**Electronic supplementary material:**

The online version of this article (10.1186/s12985-018-0934-6) contains supplementary material, which is available to authorized users.

## Background

It’s estimated that up to 7 million patients are infected with both human immunodeficiency virus (HIV) and hepatitis C virus (HCV) worldwide [1]. As HIV and HCV share similar route of transmission, serious liver disease caused by HCV has emerged as one important cause of non-AIDS associated morbidity and mortality in co-infected patients [2–4]. Eradication of HCV in co-infected patients has relation to the reduction in development of liver disease, HIV progression and mortality not related to liver disease [5]. The development of direct acting antivirals (DAAs) for treatment of HCV has been eagerly awaited to improve HCV treatment. Though numerous new DAAs are being developed, treatment of HCV in HCV/HIV co-infected patients remains complex with challenges including drug-drug interactions between HIV drugs and HCV protease inhibitors, high rates of adverse events (AEs), high pill burden and long treatment duration [6]. Sofosbuvir (SOF) which is an oral nucleotide analogue inhibitor of the HCV non-structural 5B (NS5B) polymerase has recently been approved for treatment of patients infected of HCV genotypes 1–4 [7, 8]. As SOF has minimal drug interactions with DAAs, clinical studies supporting the use of SOF in combination with other DAAs for the treatment of HCV/HIV co-infected patients were emerging. A few studies of them have performed post-hoc analyses to evaluate the efficacy and safety of SOF with or without other DAAs in patients with certain characteristics. However, a systematic review with a comprehensive comparison of the outcome data identified by the clinical studies is still not available. This study aims at summarizing the currently available data on treatment of HCV in HIV with SOF and to provide guidance in practical clinical algorithms of HCV/HIV co-infected patients’ management.

## Methods

### Searching strategy and selection of literature

Three electronic database, including PubMed, EMBASE and Cochrane databases were searched for studies. The literature search was performed using the following terms: “hepatitis C” or “HCV” or “hepacivir*”; “sofodbuvir” or “Sovaldi” or “SOF” or “GS-7977” or “PSI-7977”; “HIV” or “AIDS” or “human immunodeficiency virus”. We also performed manual search through checking the references of included studies and published narrative reviews for potentially eligible studies.

Clinical trials, case-control studies or prospective cohort studies aiming at assessing the efficacy and safety of the SOF-containing regimens in patients co-infected with HCV and HIV were included. Published or unpublished studies were enrolled if they met the following criteria: (1) the study population were co-infected with HCV and HIV; (2) interventions included SOF; (3) the main outcome measure was SVR12; (4) the studies reported the number of patients who achieved and failed to achieve SVR12; (5) the studies showed the results of safety outcome. Studies were excluded if they met any of the following criteria: (1) participants were co-infected with other virus; (2) the main outcome measure was not SVR12; (3) studies failed to report the main outcome; (4) conference abstracts without full text.

### Study selection and data extraction

Two reviewers ZK and ZDY independently selected articles potentially eligible for inclusion by screening titles, abstracts of each article. Then they tried their best to get the full texts and assessed the eligibility of the previous selected articles by reviewing the full text. After initial screening, references within the selected articles were reviewed to identify additional relevant articles. Duplicated articles with report on the same group of patients were considered once by including the most relevant or the latest article.

The two reviewers ZK and ZDY also independently extracted data using a prescribed form. The following data were extracted: first author’s name, year of publication, study design, sample size, patients’ clinical characteristics (age, sex, BMI), drug dose, treatment history and treatment duration, efficacy and safety outcomes. In case of discrepancies between the investigators during the process of article selection and data extraction, a third investigator would make the definitive decision.

Studies were through quality assessment by Jadad Scale [9]. The score contains 3 items: randomization, blinding, withdraws and dropouts. The lowest score is 0 and the highest score is 2 for the first 2 items and 1 for the third item. The total score of each study ranged from 0 to 5.

### Statistical analysis

As previous defined, SVR12 was used to estimate the efficacy. Safety outcomes included discontinuation of treatment related to therapy, AEs, serious adverse events (SAEs).

The main outcomes were expressed as dichotomous variables with 95% confidence interval (CI). Heterogeneity among the studies was evaluated by Cochrane Q test with indices *I*^2^ and *P* value. The statistical significant level was set at 0.05. If heterogeneity existed among the included studies, random effect model was used and Der Simonian and Laird method was used to calculate the pooled results [10]. Funnel plots were used to evaluate publication bias along with Egger’s statistics [11]. Subgroup analyses and meta-regression analysis were also performed in this study. All the analyses were performed using Stata (version 13.0).

## Results

### Characteristics of the included studies

**Fig. 1:**
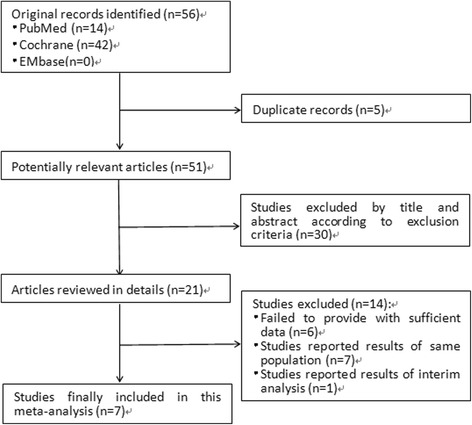
Flow diagram of study selection

Seven studies met the inclusion and exclusion criteria were finally enrolled in this meta-analysis [12–18]. Figure 1 shows the flowchart illustrating the selection procedure. In total, 56 studies were identified through electronic database and manual searching. Five studies were duplicated and then removed. The titles and abstracts of the remaining articles were then screened. Among which 51 potentially eligible studies were selected for full-text and finally seven studies were included according to the predefined criteria.

Baseline characteristics of the participants were shown in (see Additional file 1: Table S1). A total of 1167 patients aged from 18 to 75 years co-infected with HCV and HIV were enrolled in our study, in which 948 patients were males and 147 patients have received prior treatment of HCV. Overall 49 patients were infected with HCV genotype 1 and 318 patients were infected with HCV genotypes 2, 3 and 4.

### Efficacy outcomes

**Fig. 2:**
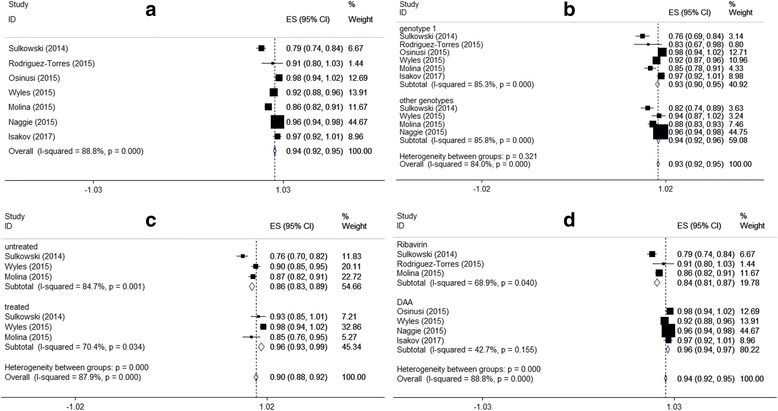
Forest plots of SVR12 based on different characteristics (**a** Forest plot of overall SVR12; **b** Forest plot of SVR12 based on HCV genotypes; **c** Forest plot of SVR12 based on treatment history; **d** Forest plot of SVR12 based on treatment therapy)

The pooled and subgroup analysis were shown in Fig. 2. Random-effect model was adopted as the *I*^2^ was over 80% (*χ*^2^ = 53.73, *P* < 0.01). The pooled SVR12 was 94.0% (95%CI: 92.0%–95.0%).

Based on treatment history, combined drugs, and HCV genotype, we subsequently performed subgroup analyses. The pooled SVR12 in patients infected with HCV genotype 1 and genotypes 2, 3 and 4 were 89.4% (95%CI: 83.3%–95.6%) and 90.3% (83.6%–96.9%), respectively. The overall test of heterogeneity between patients infected with HCV genotype 1 and other types was not significant (*P* = 0.32). The results based on treatment history showed that the SVR12 in untreated patients was 86.0% (95%CI: 83.0%–89.0%), which was lower than that in treated patients with 96.0% (95%CI: 93.0%–99.0%; *χ*^2^ = 21.39, *P* < 0.01). Considering the combination therapy, the subgroup analysis showed that heterogeneity among patients using RBV and other DAAs both were not significant. Patients treated with SOF plus other DAAs had higher SVR12 than those treated with SOF and RBV.

### Safety outcomes

**Fig. 3:**
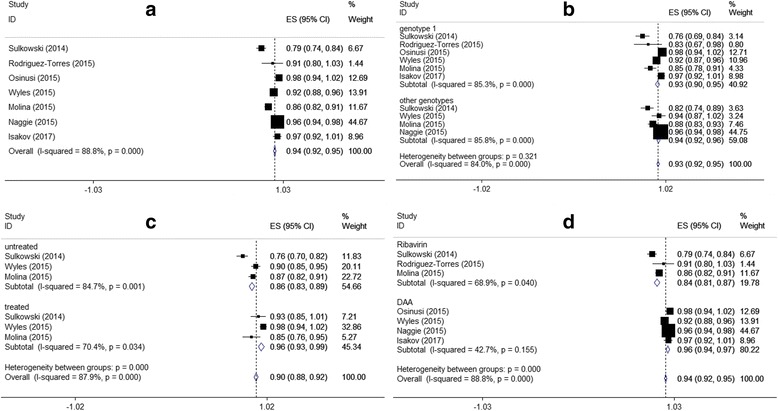
Plots of safety outcome (**a** Forest plot of discontinuation; **b** Forest plot of SAEs; **c** Forest plot of any AEs; **d** Forest plot of any AEs based on treatment therapy)

By pooling the data from five of the seven studies, we observed that the heterogeneity of incidence of discontinuation and SAEs was not significant (*χ*^2^ = 0.42, *P* = 0.52; *χ*^2^ = 4.90, *P* = 0.18). Pooled estimates of the rate of discontinuation and SAEs were 2.5% (95%CI: 1.2%–3.9%) and 2.8% (95%CI: 1.4%–4.3%), respectively (Fig. 3).

The most common AEs were fatigue, insomnia, asthenia, headache, diarrhea and nausea. Pooled results from six of the seven studies revealed high heterogeneity. The pooled incidence of any AEs was 67.8% (95%CI: 52.8%–82.8%). Significantly higher occurrence rates of AEs might be found in the therapy with RBV (89.0%, 95%CI: 86.0%–93.0%; *χ*^2^ = 55.17, *P* < 0.01) (Fig. 3).

### Publication bias

**Fig. 4:**
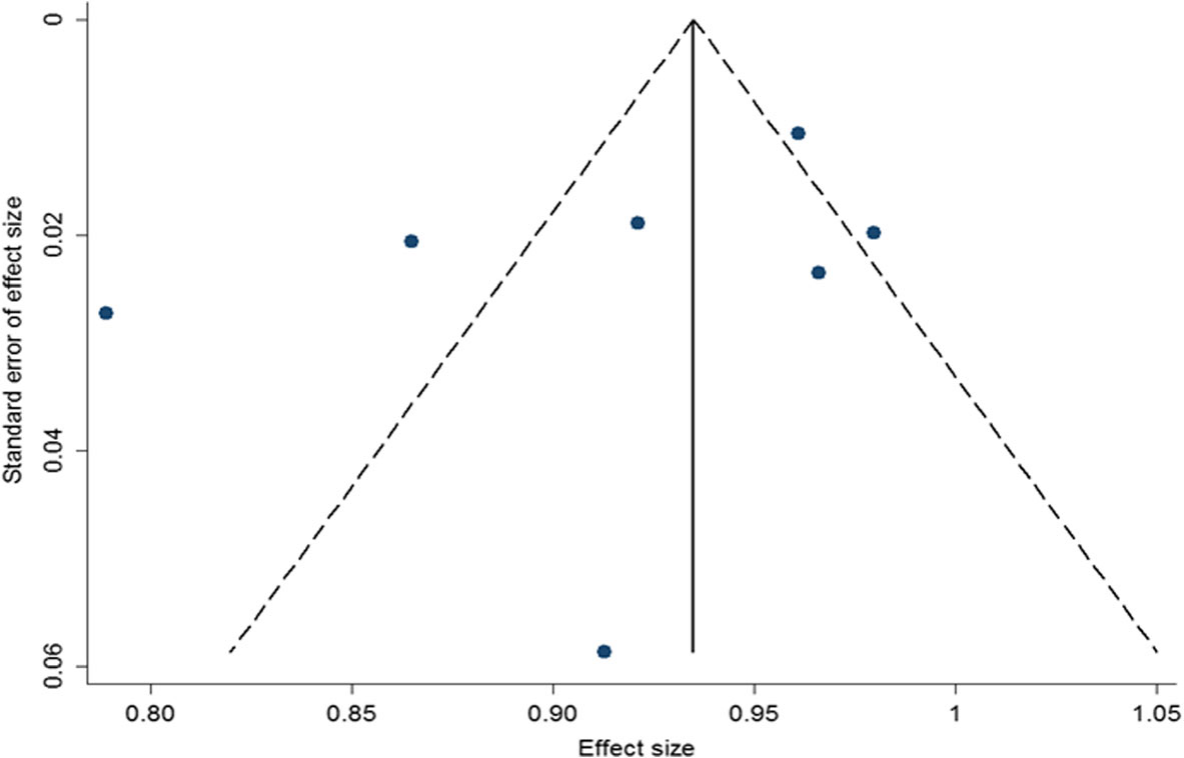
Funnel plot of SVR12

The funnel plot for SVR12 was shown in Fig. 4. Studies distributes closely within the 95% confidence interval axis, which indicated no obvious publication bias. In addition, the Egger’s test for evaluating publication bias also showed no statistical significance (*t* = − 1.22, *P* = 0.28).

### Meta-regression analysis

**Fig. 5:**
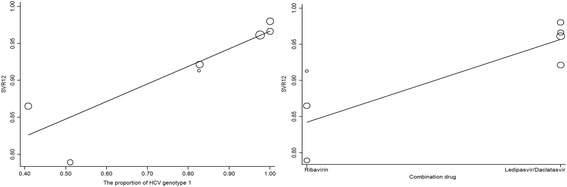
Bubble plots of SVR12

The regression analysis was performed adjusting age, sex, BMI, HCV genotype and the combination drug. Finally we found the proportion of HCV genotype 1 (*t* = 4.65, *P* < 0.01) and the combination drug (*t* = 3.91, *P* = 0.01) were with statistical significance, which could explain the heterogeneity. The Bubble plot was shown in Fig. 5.

## Discussion

In this comprehensive meta-analysis of seven studies evaluating the efficacy and safety of SOF for treatment in HCV/HIV co-infected patients, we found that the SVR12 was 94.0% (95%CI: 92.0%–95.0%) and the rate of any AEs was 67.8% (95%CI: 52.8%–82.8%).

Previous studies have summarized the drug-drug interaction between HCV and HIV treatments [19–21]. However, those reviews did not provide an overall estimation of the efficacy and safety of DAAs for treatment of HCV in HCV/HIV co-infected patients. SOF with its all HCV genotype activity and high barrier of resistance [22] is becoming an attractive target for anti-HCV treatment, while the effect in HCV/HIV co-infected patients was evaluated in some certain patients. This present study firstly attempt to review associated studies and provide a comprehensive analysis considering genotypes, treatment history, treatment regimen and other characteristics.

Although the heterogeneity was significant, it can be accounted for different combination therapies through subgroup analysis. The inconsistent conclusions in Sulkowski study [12] may be owing to its enrollment of few patients with very low CD4 cell count. In addition, in that study patients with cirrhosis (10%) and women (17%) were under presented. Subgroup analysis based on combination therapy found that patients with treatment of SOF and other DAAs showed higher SVR12 and lower occurrence of AEs than those treated with SOF and RBV. Meta-regression analysis also indicated the cause of heterogeneity.

Taking into consideration all the complex issues surrounding treatment in HCV/HIV co-infected patients, it is important to ensure that all therapy are documented and scrutinized for potential drug-drug interactions and side effects. Also it is important that patients are managed in centers with experience in managing HCV/HIV co-infected patients.

The strength of this meta-analysis lies in its exhaustive literature research, comprehensive statistical analysis and no significant evidence of publication bias. And the present study is the first research aiming at evaluating a drug in HCV/HIV co-infected patients.

This study has several limitations. First, most of the studies included in the analysis were uncontrolled trials due to the special management of HIV patients. Thus the single-arm trials limit the ability to derive definitive conclusions regarding the safety and efficacy of this regimen. Second, the original study designs slightly differing eligibility criteria of patients and treatment schedules may cofound the final results. All of these factors may lead to heterogeneity and therefore affect the estimates. Third, we did not conduct cost-effective analysis to assess the benefit and availability in clinical practice.

## Conclusion

In summary, the present meta-analysis suggests that SOF containing regimen has shown positive effect for the treatment of HCV/HIV co-infected patients, especially in those treatment-naive patients. Compared to RBV, patients treated with SOF combined with other DAAs had higher SVR12 and less AEs. Further large-scale, high quality and better designed clinical trials are needed to assess the combination of SOF and other DAAs based therapy.

## Additional file

Additional file 1: Table S1. Baseline characteristics of participants. Table S1 shows the information of selected articles and the baseline characteristics of participants. (XLS 16 kb)

## Abbreviations

AEs: Adverse events
DAAs: Direct acting antivirals
HCV: Hepatitis C virus
HIV: Human immunodeficiency virus
NS5B: Non-structural 5B
RBV: Ribavirin
SAEs: Serious adverse events
SOF: Sofosbuvir
SVR12: Sustained virological response
